# Neurological adverse events associated with antidepressants: a comprehensive 22-year analysis of the FDA adverse event reporting system

**DOI:** 10.3389/fphar.2025.1644241

**Published:** 2025-08-01

**Authors:** Qian Yu, Jingyang Yao, Enping Li, Mingkai Xia, Ziang Hu, Yun Xiao, Jianliang Huang, Mingsheng Lei

**Affiliations:** ^1^ Zhangjiajie Hospital Affiliated to Hunan Normal University, Zhangjiajie, Hunan, China; ^2^ Changsha Central Hospital, Changsha, Hunan, China; ^3^ The Key Laboratory of Carcinogenesis of the Chinese Ministry of Health, The Key Laboratory of Carcinogenesis and Cancer Invasion of the Chinese Ministry of Education, School of Basic Medicine, Cancer Research Institute, Central South University, Changsha, China; ^4^ Zhangjiajie College, Zhangjiajie, Hunan, China

**Keywords:** antidepressants, pharmacovigilance, neurological adverse events, FAERS, disproportionality analysis

## Abstract

**Introduction:**

Antidepressants are among the most commonly prescribed medications worldwide; however, comprehensive analyses of neuropsychiatric adverse events (AEs) across different drug classes and patient subgroups remain scarce.

**Methods:**

The primary objective of this study was to utilize the U.S. Food and Drug Administration’s Adverse Event Reporting System (FAERS) database to identify and characterize neurosafety signals associated with seven classes of antidepressants. Individual case safety reports involving 33 antidepressants were analyzed from 2004 to 2025, focusing on neurological AEs. The reports’ odds ratios (RORs) were calculated and presented. Kaplan-Meier methods were employed for time‐to‐event analysis, and subgroup analyses were conducted to explore patterns specific to age, gender, and drug class.

**Results:**

The database contained 127,568 neurological AEs, accounting for 33.8% of the total reports of antidepressant. Reported data were traced to 98 countries and regions, primarily from North America and Western Europe. The number, type, and severity of reported neurological AEs varied significantly by gender, age groups, and drug categories. In the adverse reaction signal analysis, a series of strong adverse reaction signals were identified, with “neonatal movement disorders” showing the strongest signal (ROR = 51.97), and serotonin syndrome signals were also prominent. Distinct signals were also identified in the analysis of various drug categories. For instance, SSRIs and SNRIs exhibited signal patterns associated with neonatal adaptation, NaSSAs displayed the strongest single signal in “motor dysarthria,” and MAOIs were associated with severe motor emergencies. NDRIs demonstrated excessive activation of the sympathetic nervous system, while the strongest signals for SARI/SMS drugs were concentrated in “visual stereotypy” and “hypoglycemic encephalopathy.” TCAs exhibited the broadest spectrum of neurological AEs. Serotonin syndrome is present in nearly every drug group. The median onset time for neurological AEs was 45 days. Significant differences were observed between drug categories, with MAOIs having the longest median onset time (91 days). Onset time was unrelated to gender but closely associated with age groups.

**Conclusion:**

Overall, this 22‐year database analysis revealed diverse patterns of neurological AEs associated with antidepressants, providing evidence to inform safe clinical decision‐making regarding drug use across populations.

## 1 Introduction

Antidepressants represent one of the most frequently prescribed drug classes worldwide, with usage rates continuing to rise across all demographic groups (Luo et al., 2020; Grace et al., 2018). The prevalence of antidepressant prescribing has increased substantially over the past two decades, driven by expanding clinical indications and growing recognition of mental health disorders. However, despite their clinical efficacy, antidepressants are associated with a broad spectrum of adverse events (AEs) that can significantly impact patient safety and quality of life (Henssler et al., 2022; Wang et al., 2024).

Antidepressant medications play a critical role in alleviating symptoms of depression; however, their use is associated with various neurological side effects, such as headaches, dizziness, tremors, sleep disturbances, and potentially severe seizures and extrapyramidal symptoms and so on (Carvalho et al., 2016; Khawam et al., 2006; Mitsch, 2013). These side effects not only impair patients’ treatment adherence but may also result in medication discontinuation and heightened clinical risks. These side effects are primarily linked to the diverse mechanisms through which antidepressants modulate the neurotransmitter system. However, these neurological risks are not confined to a particular drug class but are observed across several antidepressants, each with its distinct safety profile.

Although antidepressants from different classes exhibit substantial differences in chemical structure and target mechanisms, most medications induce a set of common neurological side effects. Headaches and dizziness are relatively prevalent during antidepressant therapy (Sharma, 2017; Edinoff et al., 2021). This may be ascribed to the drugs’ alteration of neurotransmitter balance within the central nervous system, which can potentially affect vascular regulation and directly interfere with the regulatory functions of the cerebral cortex and hypothalamus. Secondly, insomnia or somnolence are also frequent adverse reactions (Edinoff et al., 2021). As antidepressant treatment progresses, some patients may experience persistent cognitive impairment and a decline in executive function, which may be associated with long-term systemic changes and receptor adaptations (Carvalho et al., 2016; Edinoff et al., 2021).

While the aforementioned adverse reactions overlap to some extent across different drugs, specific categories display distinct neurological side effect profiles based on their primary mechanisms of action. For instance, selective serotonin reuptake Inhibitors (SSRIs) primarily cause serotonin syndrome, sexual dysfunction, and movement disorders/tremors, which are linked to excessive activation of the 5-HT system (Edinoff et al., 2021; Köhler et al., 2016; Baldwin et al., 2015; Choi et al., 2025; O'Neill-King et al., 2025). The use of serotonin–noradrenaline re-uptake inhibitors (SNRIs), in addition to causing side effects similar to SSRIs, also leads to sympathetic nervous system symptoms, such as increased blood pressure and heart rate, due to their concurrent effects on the norepinephrine system (Sharma, 2017; Santarsieri and Schwartz, 2015; Takata et al., 2019; Park et al., 2020). Moreover, tricyclic and tetracyclic antidepressants (TCAs) produce a distinct spectrum of side effects due to their non-specific multi-receptor action, including strong anticholinergic side effects (e.g., dry mouth, blurred vision, cognitive confusion), sedation, and dizziness, as well as an elevated risk of seizures (Carvalho et al., 2016; Sharma, 2017; Gopaul and Altalib, 2024). In contrast, monoamine oxidase inhibitors (MAOIs), which inhibit the degradation of neurotransmitters, often lead to severe adverse reactions, including hypertensive crises and widespread neurotransmitter dysfunction (Sharma, 2017; Alvano and Zieher, 2020; Edinoff et al., 2022). Selective noradrenaline re-uptake inhibitor (NDRIs), as norepinephrine-dopamine reuptake inhibitors, do not significantly affect the 5-HT system, resulting in notable differences in neurological adverse reactions when compared to SSRIs. They carry a reduced risk of sexual dysfunction and may cause excessive excitement, anxiety, and insomnia (Sharma, 2017; Baldwin et al., 2006).

Additionally, gender and age may also be factors contributing to different neurological adverse events. Previous studies have shown that the incidence and severity of neurological side effects associated with antidepressants may vary across different age groups and between males and females (LeGates et al., 2019; Minoc et al., 2024). For example, children and adolescents may exhibit unique patterns of adverse events compared to adults, while elderly patients may face unique risks due to polypharmacy and age-related changes in drug metabolism (Minoc et al., 2024). Gender differences in AEs suggest that gender-specific factors may influence the risk and nature of neurological side effects (LeGates et al., 2019). Additionally, some neurological adverse events may occur shortly after treatment initiation, while others may develop over weeks or months (Braund et al., 2021). Understanding the timing patterns of these adverse reactions is critical for clinicians to optimize the safety and efficacy of antidepressant use.

Although the above examples are only a few, they suffice to illustrate that the neurological adverse reaction characteristics of antidepressants share both similarities and differences. While individual clinical trials provide important safety data, they are often limited by small sample sizes, short follow-up periods, and selective patient populations that may not capture the full spectrum of neurological risks in real-world clinical practice. The U.S. Food and Drug Administration (FDA) Adverse Event Reporting System (FAERS) database contains adverse event reports, medication error reports, and product quality complaints resulting in AEs submitted to the FDA, designed to support post-marketing safety surveillance programs (Zh et al., 2023). Recent pharmacovigilance studies have demonstrated the utility of large-scale database analyses for identifying safety signals not detected in clinical trials (Yu et al., 2021; Guo et al., 2022).

To address these knowledge gaps, we conducted a comprehensive 22-year pharmacovigilance analysis of the FAERS database with the following primary objectives:

1. To systematically characterize the neurological adverse event profile of 33 antidepressants across seven pharmacological classes using robust disproportionality analysis methods.
2. To identify and quantify neurological safety signals associated with antidepressant exposure across different antidepressant classes and demographic subgroups.
3. To determine time-to-onset patterns for neurological adverse events.
4. To establish drug class-specific neurological features that may inform clinical decision-making and targeted monitoring strategies.

This study represents the largest and most comprehensive pharmacovigilance analysis of antidepressant-associated neurological adverse events to date to provide clinically relevant insights for improving patient safety and informing regulatory decision-making.

## 2 Methods

### 2.1 Data source and study design

We performed a retrospective pharmacovigilance study using individual case-safety reports (ICSRs) submitted to the FAERS database. All quarterly data files from 2004 Q1 through 2025 Q1 were downloaded from the FAERS Public Dashboard and concatenated. The study evaluates post-marketing neurological safety signals linked to antidepressant use.

### 2.2 Identification of antidepressant exposure

#### 2.2.1 Construction of the drug list

To ensure a comprehensive and clinically relevant analysis, the selection of antidepressants was conducted through a systematic, multi-stage process. Our goal was to include agents from all major pharmacological classes that are widely recognized for the treatment of depressive disorders.

First, we established a foundational framework based on established pharmacological classifications. We identified seven major classes of antidepressants from authoritative pharmacology resources: SSRIs, SNRIs, Tricyclic and Tetracyclic Antidepressants (collectively referred to as TCAs for this study); Norepinephrine-Dopamine Reuptake Inhibitor (NDRI); 5-HT_2_ Receptor Antagonist/Serotonin Modulators (SARI/SMS); Noradrenergic and Specific Serotonergic Antidepressants (NaSSAs); and Selective Noradrenaline Reuptake Inhibitor (NARI) (Procyshyn et al., 2019; Stahl, 2021).

Within each class, we selected agents that are widely recognized and frequently prescribed in major markets. The final list consists of 33 antidepressants. This curated list is not exhaustive of all agents approved by the FDA. For instance, recently approved agents with novel mechanisms such as esketamine and zuranolone, or those with very specific indications such as brexanolone for postpartum depression were not included, as their reporting profiles in FAERS may not yet be mature or comparable to established agents. Conversely, to ensure a thorough analysis of key drug classes, our list intentionally includes agents like moclobemide and reboxetine, which are not FDA-approved but are used extensively in other regions and are present in the FAERS data.

This systematic, class-driven, and evidence-based methodology was designed to mitigate selection bias. By not limiting our scope to a single country’s approved drug list and instead focusing on established pharmacological categories, we aimed to provide a more robust and globally relevant analysis of neurological AEs associated with antidepressant use. The full list of the 33 selected agents, organized by pharmacological class, is provided in Supplementary Material.

#### 2.2.2 Drug-field matching strategy

An ICSR was flagged as “suspect antidepressant exposure” when either the DRUGNAME field matched any generic, brand or synonym from Supplementary Material, using case-insensitive regular expressions.

### 2.3 Adverse-event definition

All reported reactions were coded to MedDRA version 27.1. The analysis was restricted to Preferred Terms (PTs) under the System Organ Class “Nervous system disorders”, capturing events such as seizures, movement disorders, neuropathies and altered consciousness.

### 2.4 Data cleaning and de-duplication

Duplicate ICSRs were removed to avoid multiple counting of the same case. Two or more reports were considered duplicates when the following seven variables were identical: Sex, Age, Country, EVENT_DT, Reaction (PT), Drug and Indication. Within each duplicate set, only the chronologically latest report was retained.

### 2.5 Disproportionality analysis

For every “antidepressant–neurological PT” pair, a 2 × 2 contingency table was constructed and four signal-detection algorithms were applied in Supplementary Material, We performed a disproportionality analysis using four established algorithms: the Reporting Odds Ratio (ROR), the Proportional Reporting Ratio (PRR), the Information Component (IC) from a Bayesian Confidence Propagation Neural Network (BCPNN), and the Empirical Bayes Geometric Mean (EBGM) from the Multi-item Gamma–Poisson Shrinker (MGPS) model. To ensure the statistical stability and minimize spurious signals, only drug-adverse event combinations (DECs) with three or more individual case reports were included in the disproportionality analysis.

A signal was deemed positive when at least one of the following criteria was met:

ROR lower 95% confidence limit (ROR_025_) > 1

PRR >2 and χ^2^ ≥ 4

IC lower 95% confidence limit (IC_025_) > 0.

EBGM lower 90% confidence limit (EBGM_05_) > 2

To control for false positives arising from multiple comparisons, we also calculated p-values for each pair using the Chi-square test or Fisher’s Exact Test. These p-values were subsequently adjusted using the Bonferroni correction, with a corrected p-value <0.05 indicating a highly significant association.

### 2.6 Time-to-onset (TTO) analysis

TTO was defined as the interval between therapy initiation (START_DT) and adverse-event onset (EVENT_DT). Records with missing or implausible dates were excluded. Kaplan-Meier curves were generated with the “survival” package and compared by log-rank tests (P < 0.05) across.

### 2.7 Software and data availability

All data wrangling and statistical analyses were conducted in R 4.5.0 (R Foundation for Statistical Computing). De-identified aggregate data and reproducible scripts will be deposited in an open repository after acceptance of the manuscript.

## 3 Results

### 3.1 Baseline data

**FIGURE 1:**
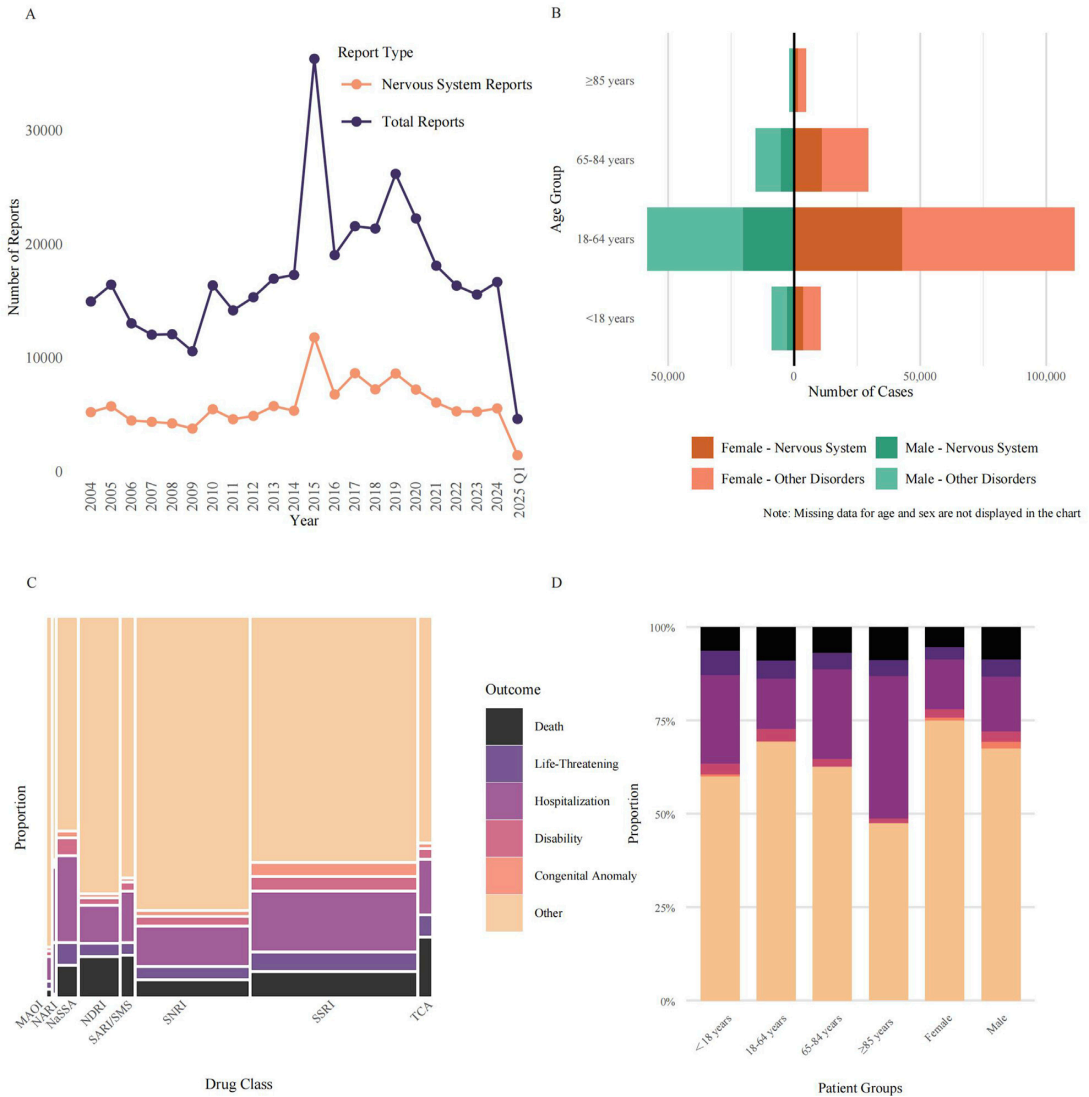
Characterization of antidepressant-associated neurological adverse events in the FAERS database. **(A)** Annual reporting trends (2004–2025) from the FAERS database for antidepressants designated as the PS drug. Lines depict the total number of annual reports versus the subset of reports classified as neurological AEs under the MedDRA SOC “Nervous System Disorders”. The final year’s data is incomplete. **(B)** Age-sex distribution of reported AEs, showing absolute report counts for males (left) and females (right) across four age groups. Bars are stratified into Neurological AEs (darker shades) and all other AEs (lighter shades). **(C)** Mosaic plot showing the proportional distribution of clinical outcomes for Neurological AEs across eight antidepressant classes. The width of each column is proportional to the report volume for each class, and the height of each colored segment represents the proportion of a specific outcome (Death, Life-Threatening, Hospitalization, Disability, Congenital Anomaly, or Other). **(D)** Proportional stacked bar chart illustrating the distribution of the six clinical outcomes for Neurological AEs, stratified by patient demographic groups. Abbreviations: AEs: Adverse Events; FAERS: FDA Adverse Event Reporting System; MAOI: Monoamine Oxidase Inhibitor; MedDRA: Medical Dictionary for Regulatory Activities; NaSSA: Noradrenergic and Specific Serotonergic Antidepressant; NARI: Selective Norepinephrine Reuptake Inhibitor; NDRI: Norepinephrine-Dopamine Reuptake Inhibitor; PS: Primary Suspect; SARI/SMS: Serotonin Antagonist and Reuptake Inhibitor/Serotonin Modulator and Stimulator; SNRI: Serotonin-Norepinephrine Reuptake Inhibitor; SOC: System Organ Class; SSRI: Selective Serotonin Reuptake Inhibitor; TCA: Tricyclic Antidepressant.

During the 22-year observation window (2004–2025) we retrieved 376,990 spontaneous FAERS reports listing one of the 33 antidepressants of interest. Of these, 127,568 (33.8%) cited at least one neurological adverse event (MedDRA SOC “Nervous system disorders”). Reporting volume showed only modest long-term growth: annual submissions rose from 14, 947 in 2004 to 16,650 in 2024, corresponding to a compound annual growth rate (CAGR) of 0.5%, data for the first quarter of 2025, comprising 4,612 reports, were excluded from this trend analysis as they did not represent a full calendar year. Neurological AEs followed a similar but slightly flatter trajectory, increasing from 5 210 to 5 542 reports over the same interval (CAGR 0.3%) from 2004 to 2024, data for the first quarter of 2025, comprising 1,421 neurological reports, were also excluded from this trend analysis. The time-course was highly non-monotonic. Both total and neurological reports declined steadily from 2004 to 2009, rebounded in 2010, and reached a pronounced apex in 2015 (36,287 total reports, 11,787 neurological reports) (Figure 1A).

Out of 376,990 total reports, Figure 1B illustrates the demographic distribution for those reports with available age and sex data. Female patients were the predominant reporters, accounting for 224,634 cases (59.6% of the total). Male patients comprised 109,958 cases (29.2%), while in 42,398 cases (11.2%), sex was not specified. Among reports where sex was specified, the number of female reporters was approximately double that of male reporters, yielding a calculated female-to-male ratio of 2.0:1. The same sex skew was evident among neurological adverse reactions, after excluding reports with missing age data, for which approximately 59,076 reports (representing 67.9% of the 87,053 nervous-system subset where sex was specified) originated from female patients.

The age-sex pyramid indicated that working-age adults (18–64 years) generated the largest share of submissions (169,527 reports; 45.0% of all cases), followed by older adults aged 65–84 years (44,894; 11.9%). Children and adolescents (<18 years) and the very old (≥85 years) together contributed only 26,297 reports, representing 7.0% of all reports. A total of 136,272 cases lacked age information, accounting for 36.1% of the total number of cases. When neurological events were expressed as a proportion of all antidepressant adverse-event reports within each demographic stratum, enrichment was still most marked in women of reproductive/middle age: these neurological reactions constituted 38.5% of reports in females aged 18–64 years versus 35.3% in age-matched males.

Across the 376,990 FAERS reports of antidepressant AEs, SSRIs accounted for the largest share (173,306 reports, 46.0%), followed by serotonin-noradrenaline reuptake inhibitors (SNRIs; 117,498, 31.2%), noradrenaline–dopamine reuptake inhibitors (NDRIs; 40,079, 10.6%) and noradrenergic and specific serotonergic antidepressants (NaSSAs; 19,472, 5.2%) (Figure 1C). Mosaic mapping of outcome frequencies showed that most reports, irrespective of class, were coded as “Other” (range 58.7% for NaSSA to 90.9% for monoamine-oxidase inhibitors, MAOIs). The proportion of fatal outcomes varied nearly twelve-fold between classes, from 1.4% for MAOIs to 15.9% for TCAs. Life-threatening events were most common with NARIs (13.3%), TCAs (5.2%) and NaSSAs (5.3%), whereas hospitalisation was particularly frequent with NaSSAs (23.2%) and SSRIs (15.9%). Congenital anomalies were rarely reported overall (1.6% for all classes) but were proportionally higher for SSRIs (2.9%). These data highlight marked inter-class differences in the spectrum and severity of reported AEs.

Stratification by patient characteristics revealed additional heterogeneity (Figure 1D). Adults aged 18–64 years generated the majority of reports but exhibited only intermediate rates of death (9.0%) and life-threatening events (4.7%). Children and adolescents (<18 years) had the highest proportion of life-threatening outcomes (6.5%) yet lower mortality (6.4%) and disability (2.8%), while the oldest patients (≥85 years) showed the greatest likelihood of hospitalisation (38.1%) and a high death rate (8.9%). Sex-stratified analyses indicated that males experienced a higher percentage of deaths (8.8% vs. 5.4%) and hospitalisations (14.7% vs. 13.2%) than females. Collectively, the proportional stacked-bar analysis underscores clinically relevant variability in outcome severity across age and sex subgroups.

**FIGURE 2:**
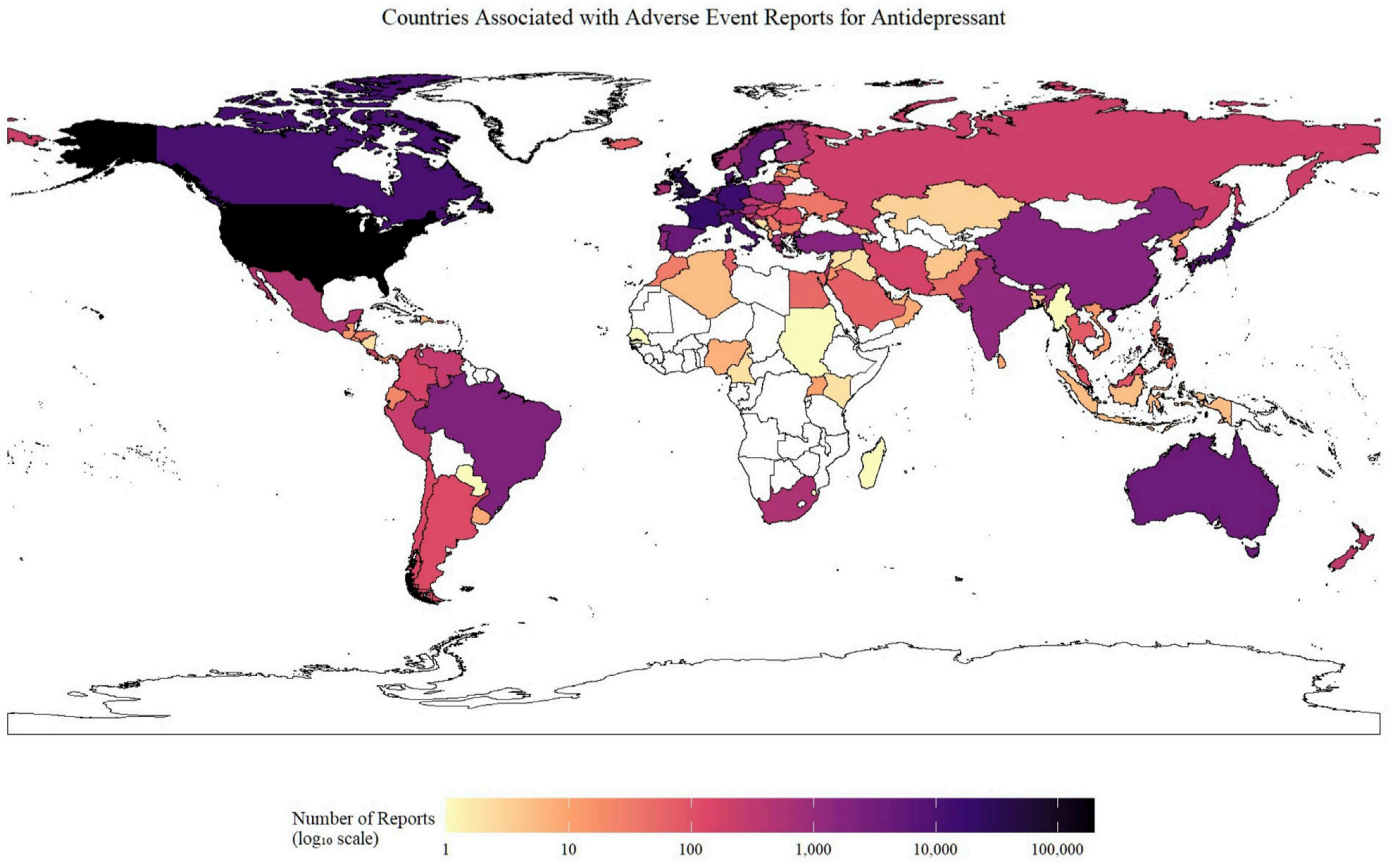
Geographical distribution of neurological adverse event reports for antidepressants submitted to the FAERS database. The choropleth map illustrates the cumulative number of neurological AE reports associated with antidepressant use submitted from each country. The color intensity corresponds to the total number of reports on a log_10_ scale, as indicated by the color bar. Countries with no submitted reports are colored white. Abbreviation: AE: Adverse Event.

Figure 2 highlights the variation in the number of reported neurological AEs associated with antidepressants across different countries. Reports were traced to 98 countries and territories. Reporting was strikingly skewed toward North America and Western Europe. The United States alone accounted for 196,066 entries, followed, at a distance, by the United Kingdom (44,362), France (19,557), Germany (15,514) and Canada (10,685). Together, these five countries generated over 75% of all neurologic safety signals linked to antidepressants in FAERS. A second tier of contributors comprised Italy (8,245),Japan (7,921) and Netherland (5,277).

### 3.2 Analysis of adverse reactions

**FIGURE 3:**
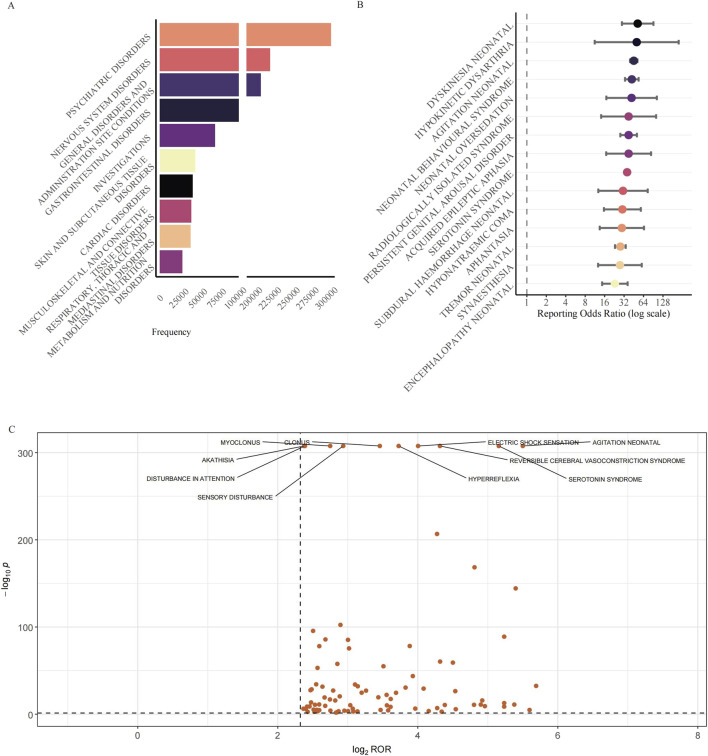
Reporting patterns and disproportionality of neurological adverse events associated with antidepressants. **(A)** Distribution of the top 10 SOCs ranked by the frequency of their associated adverse event terms associated with antidepressant use. The horizontal bar chart shows SOCs ranked by the total number of adverse event terms. A break in the x-axis is applied to accommodate the wide range of adverse event terms counts. To focus the analysis on potential pharmacological effects, SOCs not directly representing adverse reactions such as “Injury, Poisoning and Procedural Complications”, “Surgical and Medical Procedures”, “Product Issues”, and “Social Circumstances” were excluded prior to ranking. **(B)** Disproportionality analysis of the top 15 neurological AE signals. The forest plot shows signals ranked by descending ROR. Points represent the ROR, and error bars indicate the 95% CI. The dashed line at ROR = 1 represents the signal threshold. Signals were included for analysis if the lower 95% CI bound was >1 with ≥3 reports. **(C)** Volcano plot illustrating the disproportionality of individual neurological AEs across all antidepressant classes. The plot displays the log_2_(ROR) (x-axis) versus the −log_10_(p-value) (y-axis). P-values were derived from Fisher’s exact test. Data points represent AEs filtered for strong signals (case number ≥3, ROR >5, and 95% CI lower bound >1). The ten most statistically significant AEs are labelled. Abbreviations: SOCs: System Organ Classes; AE: Adverse Event; CI: Confidence Interval; FAERS: FDA Adverse Event Reporting System; PT: Preferred Term; ROR: Reporting Odds Ratio.

We analyzed the distribution of all reported adverse event terms at the System Organ Class (SOC) level (Figure 3A). The entire cohort of 376,990 reports comprised a total of 1,475,514 adverse event terms, to focus on clinically relevant events, four SOCs deemed not directly related to pharmacological reactions were excluded (“Injury, Poisoning and Procedural Complications”, “Surgical and Medical Procedures”, “Product Issues”, and “Social Circumstances”), resulting in a refined dataset of 1,321,080 adverse event terms across 23 SOCs. Figure 3A shows the top 10 SOCs ranked by the frequency of their associated adverse event terms. Among these 23 SOCs, “Psychiatric disorders” was the most frequent SOC (306,426 adverse event terms, 23.2% of all relevant terms). Strikingly, “Nervous system disorders” ranked second with 225,418 adverse event terms (17.1% of all relevant terms), highlighting the significant contribution of neurological events to the overall adverse event terms. Combined, the psychiatric and neurological SOCs accounted for over 40.0% of all relevant terms. “General disorders and administration-site conditions” formed the third largest category (213,502 adverse event terms, 16.2% of all relevant terms).

We conducted a disproportionality analysis for all retrieved drug-event pairs using the ROR, PRR, IC, and EBGM algorithms to identify potential signals. The 15 PTs with the highest ROR values are illustrated in Figure 3B. All of them remained positive when the orthogonal PRR, MGPS and BCPNN algorithms were applied, underscoring the robustness of the associations (Table 1). The strongest signal was for “Dyskinesia neonatal” (ROR = 51.97, 95% CI 29.64–91.12), followed by “Hypokinetic dysarthria” (ROR = 50.17, 95% CI 11.23–224.18) and “Agitation neonatal” (ROR = 45.24, 95% CI 39.66–51.61). Among these AEs, a cluster of neonatal-related conditions was identified, including dyskinesia neonatal, agitation neonatal, and others. Among the top 15 signals, the canonical adult toxicity, Serotonin syndrome, was not only prominent but also represented the highest absolute reporting burden, with 8,294 cases. This finding was substantiated by a robust ROR of 35.66 (95% CI 34.61–36.75). Less expected but highly disproportionate events included “Persistent genital arousal disorder” (ROR = 37.63, 28.00–50.57) and the recently characterised cognitive entity “Aphantasia” (ROR = 29.57, 13.42–65.13). The top 50 neurological AE signals, ranked by the ROR, are presented in Table 1. This includes the values for all four signal detection algorithms: ROR, PRR, EBGM, and IC.

**TABLE 1:** Disproportionality analysis of neurological adverse events associated with antidepressants in the FAERS database. Abbreviations: FAERS: FDA Adverse Event Reporting System; PT: Preferred Term; ROR: Reporting Odds Ratio; PRR: Proportional Reporting Ratio; EBGM: Empirical Bayes Geometric Mean; IC: Information Component.

PT	Case reports	ROR(95%Cl)	PRR(chi-square value)	EBGM(EBGM05)	IC(IC025)
Dyskinesia neonatal	29	51.97 (29.64–91.12)	51.96 (608.81)	22.41 (14)	4.49 (3.82)
Hypokinetic dysarthria	4	50.17 (11.23–224.18)	50.17 (82.62)	22.07 (6.31)	4.46 (2.82)
Agitation neonatal	488	45.24 (39.66–51.61)	45.23 (9585.61)	21.09 (18.89)	4.4 (4.24)
Neonatal behavioural syndrome	139	42.19 (33.11–53.74)	42.18 (2634.92)	20.42 (16.67)	4.35 (4.05)
Neonatal oversedation	10	41.81 (16.99–102.9)	41.81 (188.69)	20.33 (9.57)	4.35 (3.26)
Persistent genital arousal disorder	88	37.63 (28–50.57)	37.63 (1568.88)	19.31 (15.08)	4.27 (3.9)
Acquired epileptic aphasia	12	37.63 (16.91–83.76)	37.63 (213.94)	19.31 (9.89)	4.27 (3.29)
Radiologically isolated syndrome	8	37.63 (14.12–100.26)	37.63 (142.63)	19.31 (8.51)	4.27 (3.08)
Serotonin syndrome	8294	35.66 (34.61–36.75)	35.47 (143064.09)	18.74 (18.28)	4.23 (4.19)
Subdural haemorrhage neonatal	9	30.79 (12.76–74.3)	30.79 (142.66)	17.38 (8.32)	4.12 (3.01)
Hyponatraemic coma	16	30.1 (15.6–58.09)	30.1 (250.11)	17.17 (9.9)	4.1 (3.26)
Aphantasia	11	29.57 (13.42–65.13)	29.57 (170.02)	17 (8.78)	4.09 (3.08)
Tremor neonatal	184	28.04 (23.16–33.93)	28.03 (2748.78)	16.49 (14.06)	4.04 (3.79)
Synaesthesia	11	27.6 (12.67–60.08)	27.6 (162.66)	16.34 (8.52)	4.03 (3.03)
Encephalopathy neonatal	30	23.04 (14.63–36.29)	23.04 (392.29)	14.67 (10.03)	3.87 (3.26)
Somnolence neonatal	69	22.58 (16.75–30.43)	22.58 (889.33)	14.49 (11.28)	3.86 (3.45)
Neonatal epileptic seizure	6	22.58 (8.21–62.12)	22.58 (77.33)	14.49 (6.21)	3.86 (2.56)
Perinatal stroke	12	20.53 (10.16–41.47)	20.53 (144.22)	13.63 (7.57)	3.77 (2.83)
Reversible cerebral vasoconstriction syndrome	587	19.91 (18.01–22)	19.9 (6892.14)	13.36 (12.29)	3.74 (3.6)
Tethered cord syndrome	74	19.89 (15.01–26.36)	19.89 (868.51)	13.36 (10.55)	3.74 (3.35)
Neonatal seizure	260	19.34 (16.65–22.46)	19.34 (2986.27)	13.11 (11.57)	3.71 (3.51)
Hypokinesia neonatal	8	18.81 (8.05–43.96)	18.81 (89.96)	12.88 (6.33)	3.69 (2.57)
Morvan syndrome	3	18.81 (4.71–75.23)	18.81 (33.74)	12.88 (4.04)	3.69 (1.98)
Sympathomimetic effect	38	16.82 (11.48–24.66)	16.82 (390.81)	11.93 (8.67)	3.58 (3.05)
Sympathicotonia	4	16.72 (5.15–54.31)	16.72 (40.94)	11.89 (4.44)	3.57 (2.07)
Electric shock sensation	579	16.04 (14.55–17.68)	16.03 (5722.78)	11.54 (10.64)	3.53 (3.39)
Opsoclonus myoclonus	60	15.15 (11.23–20.45)	15.15 (565.44)	11.09 (8.63)	3.47 (3.05)
Persistent postural-perceptual dizziness	8	15.05 (6.63–34.17)	15.05 (74.96)	11.04 (5.56)	3.46 (2.36)
Drug withdrawal headache	110	14.73 (11.82–18.36)	14.73 (1011.76)	10.87 (9.04)	3.44 (3.13)
Rabbit syndrome	43	14.07 (9.91–19.97)	14.07 (379.99)	10.51 (7.84)	3.39 (2.9)
Hyperreflexia	864	13.23 (12.24–14.3)	13.22 (7223.39)	10.04 (9.41)	3.33 (3.22)
Action tremor	36	12.78 (8.76–18.65)	12.78 (291.79)	9.79 (7.14)	3.29 (2.76)
Amimia	26	12.08 (7.77–18.79)	12.08 (200)	9.39 (6.49)	3.23 (2.61)
Oculocephalogyric reflex absent	12	11.88 (6.21–22.74)	11.88 (90.9)	9.27 (5.39)	3.21 (2.32)
Sensory overload	34	11.63 ( 7.92–17.09)	11.63 (252.37)	9.12 (6.61)	3.19 (2.65)
Adrenergic syndrome	15	11.52 (6.46–20.54)	11.52 (110.32)	9.05 (5.58)	3.18 (2.37)
Poor sucking reflex	88	11.34 (8.94–14.39)	11.34 (637.58)	8.95 (7.33)	3.16 (2.82)
Lenticulostriatal vasculopathy	6	11.29 (4.53–28.11)	11.29 (43.28)	8.91 (4.15)	3.16 ( 1.93)
Clonus	573	10.98 (10–12.05)	10.97 (4021.31)	8.72 (8.07)	3.12 (2.99)
Decerebrate posture	31	10.7 (7.18–15.95)	10.7 (212.29)	8.55 (6.13)	3.1 (2.53)
Postictal headache	7	10.54 (4.56–24.36)	10.54 (47.2)	8.45 (4.19)	3.08 (1.94)
Hyponatraemic seizure	47	9.51 (6.9–13.09)	9.51 (285.66)	7.79 (5.96)	2.96 (2.5)
Thunderclap headache	44	9.1 (6.55–12.65)	9.1 (255.38)	7.52 (5.71)	2.91 (2.44)
Stiff person syndrome	59	8.78 (6.61–11.65)	8.78 (329.6)	7.3 (5.76)	2.87 (2.46)
Periodic limb movement disorder	64	8.54 (6.51–11.2)	8.54 (347.25)	7.15 (5.69)	2.84 (2.45)
Coma neonatal	5	8.18 (3.11–21.52)	8.18 (25.89)	6.9 (3.07)	2.79 (1.49)
Basilar migraine	11	8.12 (4.23–15.57)	8.12 (56.46)	6.85 (3.97)	2.78 (1.87)
Cerebral vasoconstriction	150	8.08 (6.77–9.63)	8.08 (765.56)	6.83 (5.89)	2.77 (2.52)
Decorticate posture	19	8.03 (4.9–13.18)	8.03 (96.42)	6.8 (4.49)	2.76 (2.06)
Postictal state	171	7.99 (6.78–9.43)	7.99 (862.97)	6.77 (5.9)	2.76 (2.52)

To visualize the breadth and relative strength of all neurological disproportionalities, we plotted the continuity–corrected reporting odds ratio on a log_2_ scale against the–log_10_ of the exact Fisher P value for every preferred term that met the basic screen (a>=3, RORL >1, ROR >5). The resulting volcano plot comprised 91 nervous-system PTs (Figure 3C). The bulk of the points formed a dense cloud centred on log_2_ROR values of approximately 2.5–3.0 (an equivalent ROR of approximately 6–8) and–log_10_P values of approximately 5–15, indicating a wide array of moderate yet statistically secure signals. Ten PTs with the greatest statistical weight were labelled for ease of reference. Among them, agitation neonatal (a = 488) occupied the extreme right of the plot with the largest effect size (ROR = 45.24, 95% CI 39.66–51.61; log_2_ROR = 5.50) and an essentially infinitesimal P value (a–log_10_P value of approximately 308). Serotonin syndrome, the numerically dominant PT (a = 8,294), exhibited a similarly high effect (ROR = 35.66, 34.61–36.75; log_2_ROR = 5.16). Reversible cerebral vasoconstriction syndrome (a = 587) showed a large excess of reporting (ROR = 19.92, 18.02–22.00; log_2_ROR = 4.32; a–log_10_P value of approximately 308). Electric shock sensation (a = 579) yielded ROR = 16.05 (14.56–17.68; log_2_ROR = 4.00). Hyperreflexia (a = 864) was characterised by ROR = 13.23 (12.25–14.30; log_2_ROR = 3.73). Together with the forest plot (Figure 3B), which focuses on the fifteen largest point estimates, the volcano plot provides a complementary panoramic view, revealing not only the very top signals but also the broader context in which they reside.

### 3.3 Subgroup analysis

**FIGURE 4:**
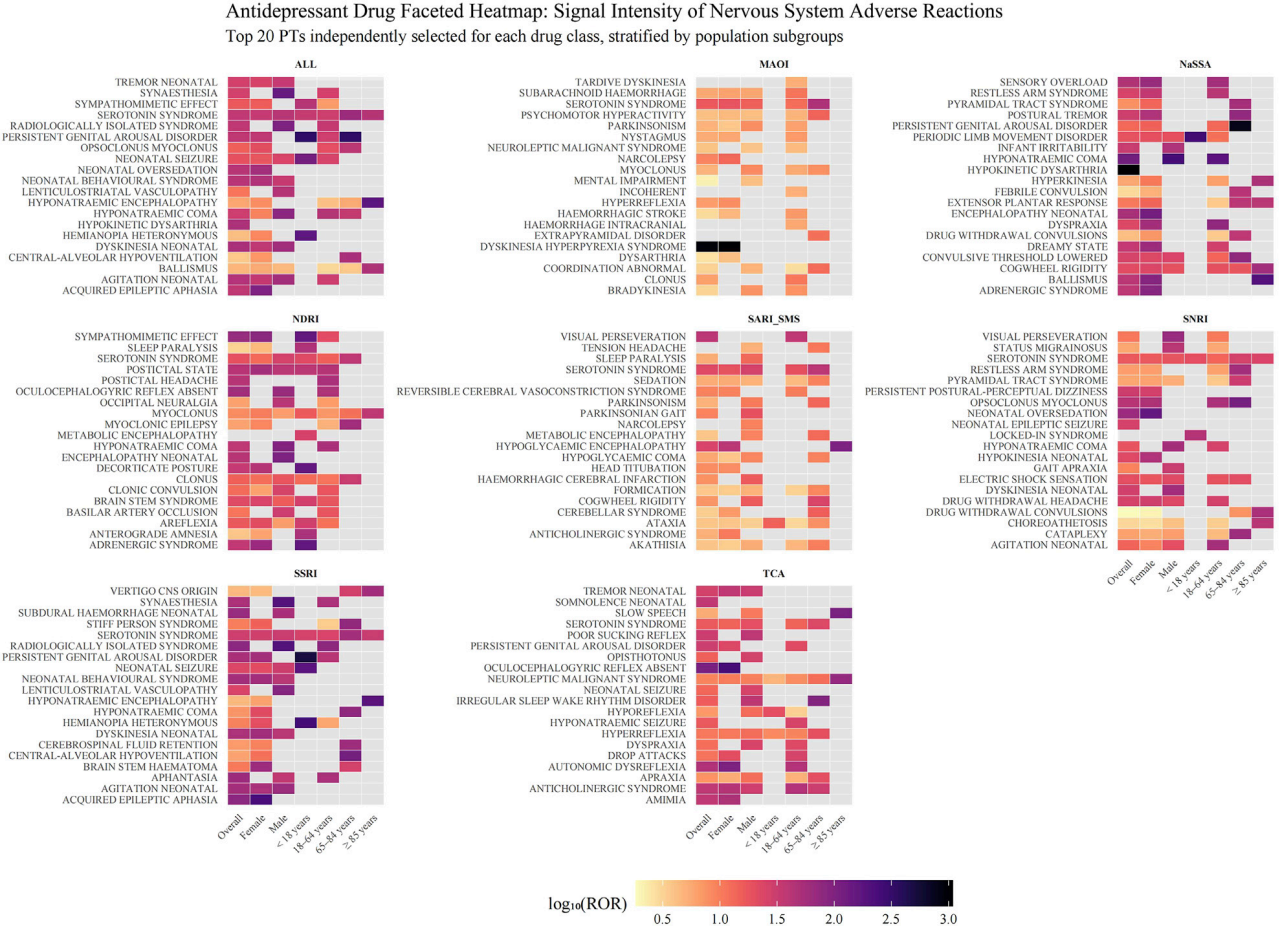
Disproportionality analysis of neurological adverse events for different classes of antidepressants. The heatmap displays signals of disproportionate reporting for neurological adverse events across nine classes of antidepressants, stratified by population subgroups. Each facet represents a distinct antidepressant class. The y-axis lists the top 20 neurological adverse events (PT level) for each class, selected based on the highest ROR observed in any subgroup. The x-axis shows the overall population and subgroups defined by sex and age. The color intensity of each tile corresponds to the signal strength, quantified as the base-10 logarithm of the ROR (log_10_(ROR)). Darker shades indicate a stronger signal. Grey tiles indicate that no signal was detected for that specific drug-event-subgroup combination, as the data did not meet the threshold criteria. The NARI class was excluded from this analysis due to an insufficient number of adverse event terms. Abbreviations: ALL: All included Antidepressant Classes Combined; FAERS: FDA Adverse Event Reporting System; MAOI: Monoamine Oxidase Inhibitor; NARI: Selective Norepinephrine Reuptake Inhibitor; NaSSA: Noradrenergic and Specific Serotonergic Antidepressant; NDRI: Norepinephrine-Dopamine Reuptake Inhibitor; PT: Preferred Term; ROR: Reporting Odds Ratio; SARI/SMS: Serotonin Antagonist and Reuptake Inhibitor/Serotonin Modulator and Stimulator; SNRI: Serotonin-Norepinephrine Reuptake Inhibitor; SSRI: Selective Serotonin Reuptake Inhibitor; TCA: Tricyclic Antidepressant.

Figure 4 shows that for each pharmacological class, the twenty neurological PTs that generated the largest disproportionality signals, colour intensity represents the log10-transformed reporting-odds ratio (log10 ROR) and panels are further split by sex and age. The NARI class was excluded from this analysis due to an insufficient number of reports. For SSRIs and SNRIs, a pattern of signals related to neonatal adaptation was observed, including “agitation neonatal”, “dyskinesia neonatal”, and “neonatal seizure/oversedation”. NaSSAs produced the most intense single signals of the entire analysis: “hypokinetic dysarthria” (log10 ROR = 3.02 overall panel) and “ballismus” (peak 2.32 in groups aged ≥85). They were also responsible for the highest risk estimate for “hyponatraemic coma” (2.40 in males). MAOIs showed a unique enrichment for severe motor emergencies, most notably “dyskinesia hyperpyrexia syndrome” (log10 ROR = 2.97 overall; 3.03 in females), and were the class with prominent intracranial haemorrhage‐related PTs. NDRIs were characterised by sympathetic over-activation (“sympathomimetic effect”, log10 ROR = 1.83 overall), whereas SARI/SMS agents clustered around “visual perseveration” (log10 ROR = 1.60 overall) and “hypoglycaemic encephalopathy” (log10 ROR = 1.40 overall). TCAs displayed the widest PT spectrum, the highest signals were confined to rare movement disorders (“oculocephalogyric reflex absent”, 2.39 in females).

“Serotonin syndrome” appeared in almost every drug group (except NaSSA). Subgroup faceting highlighted systematic sex differences, over half of class-specific PTs had higher log10 RORs in males.

### 3.4 Time to onset analysis

**FIGURE 5:**
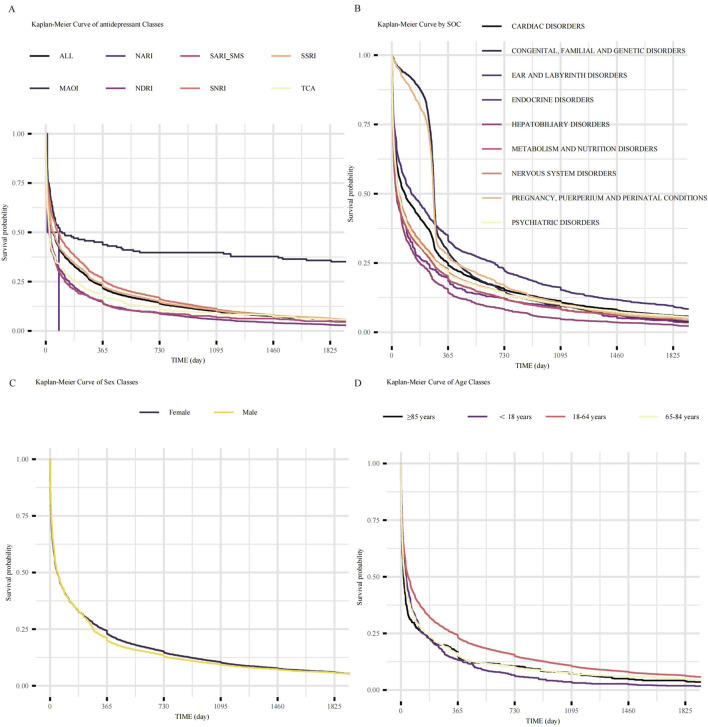
Time-to-onset analysis of adverse events following antidepressant initiation. **(A)** Kaplan-Meier curves showing the time-to-onset of neurological AEs, stratified by antidepressant class. Each line represents a distinct class as indicated in the key. **(B)** Kaplan-Meier curves showing the time-to-onset of AEs stratified by SOCs for all antidepressants combined. **(C)** Kaplan-Meier curves comparing the time-to-onset of neurological AEs between female and male patients. **(D)** Kaplan-Meier curves comparing the time-to-onset of neurological AEs across four distinct age groups. Abbreviations: AEs: adverse events; ALL: All included Antidepressant Classes Combined; FAERS: Food and Drug Administration Adverse Event Reporting System; MAOI: Monoamine Oxidase Inhibitor; NARI: Selective Norepinephrine Reuptake Inhibitor; NaSSA: Noradrenergic and Specific Serotonergic Antidepressant; NDRI: Norepinephrine-Dopamine Reuptake Inhibitor; SARI/SMS: Serotonin Antagonist and Reuptake Inhibitor/Serotonin Modulator and Stimulator; SNRI: Serotonin-Norepinephrine Reuptake Inhibitor; SOC: System Organ Class; SSRI: Selective Serotonin Reuptake Inhibitor; TCA: Tricyclic Antidepressant.

We analyzed the outcomes of AEs of different groups. The longest median time to onset was observed for MAOIs of all drug groups (91 days) (Figure 5A). Kaplan-Meier analysis of all included neurological AE reports indicated a median time-to-onset of approximately 45 days (Figure 5B). Notably, the onset profile of neurological AEs was not significantly different from that of endocrine disorders (p = 0.05) (Figure 5B).

The influence of demographic factors on AEs onset was also examined. No significant difference in time-to-onset was observed between females and males (log-rank p = 0.21; Figure 5C). However, age significantly impacted the AEs onset time (overall log-rank p < 0.05; Figure 5D). Senior patients (≥85 years) experienced the earliest AEs onsets (median time to onset 16 days), followed by the 65–84 years age group (median time to onset 24 days), then the <18 years age group (median time to onset 32 days). AEs onset was progressively delayed in 18–64 age groups (median time to onset 45 days). Pairwise comparisons revealed that the 18–64 years age stratum differed significantly from all other groups (vs. < 18 years, adjusted. p < 0.05; vs. 65–84 years, adjusted. p < 0.05; vs. ≥ 85 years, adjusted. p < 0.05). In contrast, no significant differences were observed in comparisons among the other age strata.

## 4 Discussion

This comprehensive 22-year pharmacovigilance analysis represents the largest systematic evaluation of antidepressant-associated neurological adverse events to date, encompassing 127,568 reports across 33 medications and seven pharmacological classes. Our findings reveal distinct drug class-specific neurological risk profiles, significant demographic variations, and temporal patterns. Our findings reveal several important patterns that have significant implications for clinical practice, regulatory decision-making, and our understanding of antidepressant neurotoxicity.

### 4.1 Key neurological safety signals

The most striking finding of our analysis is the predominance of neonatal neurological adverse events among the strongest disproportionality signals. Seven of the top 15 signals involved neonatal adaptation phenomena, with effect sizes exceeding ROR values of 23–52. This pattern aligns with recent comprehensive evidence from multiple large-scale studies. A 2023 Mayo Clinic analysis of 1,014 SSRI-exposed infants found poor neonatal adaptation incidence rates of approximately 30% with third-trimester exposure (Brumbaugh et al., 2023), while a Swedish population study of over 1.2 million children demonstrated elevated risks of neonatal seizures and epilepsy in SSRI/SNRI-exposed offspring (Viktorin et al., 2017). Another survey indicated a correlation between the use of antidepressants by mothers in late pregnancy and poor adaptation in newborns (e.g., feeding difficulties, excessive sleepiness, abnormal muscle tone, etc.) (Källén, 2004).

Compared with other types of antidepressants (such as monoamine oxidase inhibitors, whose mortality rate is only 1.4%), the mortality rate of tricyclic antidepressants is significantly higher (15.9%). This reflects the severe toxicity of tricyclic drugs when used in excessive amounts. The therapeutic index of tricyclic drugs is narrow, and excessive use may lead to life-threatening cardiac toxicity and central nervous system depression, thereby increasing the risk of death (Kerr et al., 2001; Woolf et al., 2007). This safety issue has led to a decrease in the usage of tricyclic drugs, while other types of drugs such as SSRIs and SNRIs have dominated due to their safer excessive usage situations.

Serotonin syndrome emerged as the most frequently reported serious neurological adverse event (8,294 cases) with a robust signal strength (ROR = 35.66), confirming its role as a major safety concern across multiple antidepressant classes (Boyer and Shannon, 2005; Francescangeli et al., 2019). The high absolute burden of serotonin syndrome cases emphasizes the importance of clinical vigilance, particularly when combining serotonergic medications or in patients with predisposing factors (Isbister et al., 2007).

The identification of unexpected neurological signals, including persistent genital arousal disorder (ROR = 37.63) and aphantasia (ROR = 29.57), represents novel pharmacovigilance findings that complement recent regulatory acknowledgments.The European Medicines Agency’s 2019 decision to include warnings about persistent sexual dysfunction following SSRI/SNRI use, recognizing that dysfunction can persist after treatment withdrawal, provides regulatory context for our persistent genital arousal disorder findings. In addition, a study have suggested that antidepressants may indirectly alter brain perception and cognitive function by affecting the serotonin system, which may be related to the occurrence of aphantasia (Dos Santos et al., 2018).

### 4.2 Drug class-specific neurological profiles

The predominance of neonatal adaptation syndromes with SSRIs and SNRIs aligns with recent mechanistic understanding of serotonin’s role in neurodevelopment. Contemporary research suggests that neonatal exposure to elevated serotonin levels during critical developmental periods disrupts normal neurotransmitter homeostasis, leading to the withdrawal-like symptoms observed in poor neonatal adaptation syndrome (Moses-Kolko et al., 2005). Recent dose-response studies have strengthened this mechanistic understanding, showing clear relationships between maternal antidepressant doses and neonatal complication severity (Brumbaugh et al., 2023).

NaSSAs demonstrated the highest signal intensities overall in our analysis, particularly for movement disorders such as hypokinetic dysarthria and ballismus. This pattern may reflect the complex pharmacology of agents like mirtazapine, which blocks multiple receptors including histamine H1, α2-adrenergic, and various serotonin receptor subtypes. The association with hyponatremic coma also aligns with known risks of syndrome of inappropriate antidiuretic hormone secretion with mirtazapine (Nagashima et al., 2022).

MAOIs demonstrated a unique enrichment for severe motor emergencies, most notably dyskinesia-hyperpyrexia syndrome. This finding is consistent with MAOIs’ complex effects on multiple neurotransmitter systems and their potential for serious drug interactions (MAOI, 2023; Asano et al., 2023). The motor complications associated with MAOIs may reflect their effects on dopaminergic pathways and their interaction with tyramine-containing substances.

TCAs exhibited the widest spectrum of neurological preferred terms, consistent with their non-selective mechanism of action affecting multiple neurotransmitter systems and receptors (Khalid and Waseem, 2025). However, contrary to expectations based on clinical literature, we did not observe prominent seizure signals for TCAs in our analysis, which may reflect under-reporting, confounding by indication, or the influence of dosing patterns in real-world practice.

NDRIs (bupropion) showed characteristic sympathomimetic effects, reflecting their unique dopamine and norepinephrine reuptake inhibition without significant serotonergic activity (Protti et al., 2020). This profile distinguishes NDRIs from other antidepressant classes and may explain their lower propensity for certain side effects like sexual dysfunction.

### 4.3 Demographic patterns and clinical implications

The consistent 2:1 female-to-male ratio observed across neurological adverse events aligns with recent large-scale epidemiological studies. A 2024 UK Biobank analysis of 222,121 adults confirmed persistent sex differences in antidepressant adverse event susceptibility, with women showing higher baseline risks for most neurological complications (Bansal et al., 2022). Women typically exhibit higher plasma concentrations of most antidepressants due to differences in body composition, hepatic metabolism, and hormonal influences on drug clearance (Franconi et al., 2007; Zucker and Prendergast, 2020). These findings support recent calls for sex-specific dosing considerations and monitoring protocols (Dragioti et al., 2019).

Age-stratified analysis provided valuable clinical insights. The study found that women aged 18–64 years exhibited the highest incidence of neurological adverse events—38.5% compared to 35.3% in age-matched men—a finding with significant implications for treatment planning. Older patients (≥85 years; 32.4%) exhibited a lower proportion of neurological adverse events, which may be attributed to competing risks from other organ systems or different reporting patterns in this population. Additionally, older patients were more likely to be hospitalized for neurological adverse events, reflecting increased vulnerability in this cohort. Conversely, although children and adolescents exhibited fewer fatal outcomes, they experienced a higher incidence of life-threatening adverse events, which may be due to developmental differences in drug metabolism and unique risks in this demographic (Minoc et al., 2024; Hämäläinen et al., 2022; Clinic, 2022). These findings underscore the necessity of tailored monitoring and dosing strategies for different age groups to mitigate the risk of adverse events.

The geographical distribution of reports, with 75% originating from five developed countries (US, UK, France, Germany, Canada), highlights potential reporting disparities and the need for enhanced pharmacovigilance infrastructure in other regions. This skewed distribution may limit the generalizability of findings to diverse global populations with different genetic backgrounds, healthcare systems, and prescribing patterns.

### 4.4 Temporal patterns and clinical monitoring

Our time-to-onset analysis revealed important temporal patterns that may inform clinical monitoring strategies. The overall median time-to-onset for neurological adverse events was 45 days, indicating that most complications occur within the first few months of treatment, emphasizing the importance of close monitoring during the initial treatment period. Significant differences were observed between drug categories, with MAOIs exhibiting the longest median onset time (91 days), which may reflect their gradual dose escalation regimen, delayed therapeutic effects, or the time required for drug interactions to manifest (Van den Eynde et al., 2023; Suchting et al., 2021). Onset time was unrelated to gender but closely associated with age groups. Patients aged ≥85 years experienced the earliest median onset time (16 days), followed by the 65–84 age group (24 days), indicating increased susceptibility in the elderly population (Coupland et al., 2011). This pattern may reflect age-related changes in drug metabolism, increased comorbidities, or drug interactions (Alemayehu et al., 2025; Zhou et al., 2023). The delayed onset in working-age adults (18–64 years: 45 days) may indicate better physiological reserves or different treatment adherence patterns.

### 4.5 Strengths and limitations

The findings of this study are grounded in an analysis of unprecedented scale and methodological rigor. Our analysis represents the largest class-wide survey of antidepressant adverse events to date, interrogating the entire public FAERS archive from Q1 2004 to Q1 2025. This comprehensive dataset, encompassing 376,990 reports for 33 distinct antidepressants, enabled robust, direct cross-class comparisons. To ensure the reliability of our findings and limit false positives, we applied a stringent signal detection strategy, retaining only those signals deemed positive by four orthogonal disproportionality algorithms (ROR, PRR, MGPS and BCPNN). Furthermore, we moved beyond mere signal detection by performing fine-grained stratification by sex, age, drug class, and clinical outcome. This approach not only highlighted vulnerable subgroups, such as neonates for SSRIs/SNRIs, but also provided crucial context on the severity of adverse events, including death, hospitalization, and disability.

Several caveats temper the interpretation of our findings. First, FAERS is a spontaneous‐reporting system devoid of exposure denominators; hence the reporting-odds ratios we used quantify disproportionality rather than absolute or relative risk. Second, the database is subject to substantial reporting biases: under-reporting obscures the true event burden, whereas notoriety and stimulated reporting may inflate signals for well-publicised toxicities such as serotonin syndrome or neonatal withdrawal. Third, individual case narratives are often incomplete, lacking uniform data on age, comorbidities, disease severity or concomitant medicines—variables that can confound neurological outcomes and that we could not systematically adjust for. Fourth, establishing definitive causality is impossible based on SRS data alone, an ambiguity particularly pronounced for the prominent neonatal signals we identified. The structure of FAERS reports often precludes distinguishing between adverse events arising from *in utero* exposure, postnatal exposure via breastfeeding, or direct infant administration, thus obscuring the precise exposure pathway and underlying mechanism. Finally, the dataset is geographically skewed: five high-income countries (led by the United States) generated more than 80% of all reports, so detected signals may reflect local prescribing customs, reporting cultures or population-specific pharmacogenetics and may not be generalisable to low- and middle-income settings. Collectively, these constraints indicate that the present results are hypothesis-generating and require confirmation in analytical pharmaco-epidemiological studies with controlled exposure data and broader geographic representation.

## 5 Conclusion

In conclusion, antidepressants are associated with a range of neurological adverse events that vary across drug classes, demographic groups, and the timing of onset. The findings of this study highlight the importance of personalized treatment strategies, especially when prescribing antidepressants to vulnerable populations. Clinicians should be aware of the potential risks associated with antidepressant therapy, particularly those related to neurological side effects, and implement proactive monitoring and management strategies. Further research is needed to confirm these safety signals and explore the underlying mechanisms behind these demographic and timing differences. In the meantime, more intensive studies on other systemic organ classes are warranted in the future to comprehensively examine the safety profiles of antidepressants.

## Data Availability

The original contributions presented in the study are included in the article/Supplementary Material, further inquiries can be directed to the corresponding authors.
