# Supplementary material for: Neurological adverse events associated with antidepressants: a comprehensive 22-year analysis of the FDA adverse event reporting system
Source: Front Pharmacol. 2025 Aug 1;16:1644241. doi: 10.3389/fphar.2025.1644241 (PMC12354350; doi:10.3389/fphar.2025.1644241)
Supplement: Supplementary file 1 [file DataSheet1.docx]

|  | Antidepressants | All Other Drugs | Total |
| --- | --- | --- | --- |
| Adverse event | *a* | *b* | *a+b* |
| All other adverse events | *c* | *d* | *c+d* |
| Total | *a+c* | *b+d* | *a+b+c+d* |

1. **2 × 2 contingency table and four signal-detection algorithms**

$$ROR =\frac{\frac{a}{c}}{\frac{b}{d}}=\frac{ad}{bc}$$

$$PRR=\frac{\frac{a}{a+b}}{\frac{c}{c+d}}$$

$$IC=\log_{2} \frac{a\left( a+b+c+d \right)}{\left( a+b \right)\left( a+c \right)}$$

$$EBGM=\frac{a\left( a+b+c+d \right)}{\left( a+b \right)\left( a+c \right)}$$

1. **Antidepressant classes included in the FAERS analysis**

| Pharmacological class (abbreviation) | Agents analysed |
| --- | --- |
| Selective serotonin re-uptake inhibitors (SSRIs) | Fluoxetine; Paroxetine; Sertraline; Citalopram; Escitalopram; Fluvoxamine; Vilazodone |
| Serotonin–noradrenaline re-uptake inhibitors (SNRIs) | Venlafaxine; Desvenlafaxine; Levomilnacipran; Duloxetine; Milnacipran |
| Norepinephrine–dopamine re-uptake inhibitor (NDRI) | Bupropion |
| 5-HT₂ receptor antagonist / serotonin modulators (SARI/SMS) | Trazodone; Nefazodone; Vortioxetine |
| Noradrenergic & specific serotonergic antidepressants (NaSSAs) | Mirtazapine; Mianserin |
| Tricyclic & tetracyclic antidepressants (TCAs) | Amitriptyline; Doxepin; Protriptyline; Maprotiline†; Nortriptyline; Amoxapine; Desipramine; Clomipramine; Trimipramine; Imipramine |
| Monoamine oxidase inhibitors (MAOIs) | Phenelzine; Selegiline; Moclobemide; Isocarboxazid; Tranylcypromine |
| Selective noradrenaline re-uptake inhibitor (NARI) | Reboxetine |
